# Tracking Chronic Disease and Risk Behavior Prevalence as Survey Participation Declines: Statistics From the Behavioral Risk Factor Surveillance System and Other National Surveys

**Published:** 2008-06-15

**Authors:** Mansour Fahimi, Michael Link, Ali Mokdad, Deborah A Schwartz, Paul Levy

**Affiliations:** Marketing Systems Group. At the time of the study, Dr Fahimi was affiliated with RTI International, Rockville, Maryland; Division of Adult and Community Health, National Center for Chronic Disease Prevention and Health Promotion, Centers for Disease Control and Prevention, Atlanta, Georgia; Division of Adult and Community Health, National Center for Chronic Disease Prevention and Health Promotion, Centers for Disease Control and Prevention, Atlanta, Georgia; RTI International, Rockville, Maryland; Research Triangle Institute, Cary, North Carolina

## Abstract

**Introduction:**

Response rates for the Behavioral Risk Factor Surveillance System (BRFSS) have declined in recent years. The response rate in 1993 was approximately 72%; in 2006, the response rate was approximately 51%. To assess the impact of this decline on the quality of BRFSS estimates, we compared selected health and risk factor estimates from BRFSS with similar estimates from the National Health Interview Survey (NHIS) and the National Health and Nutrition Examination Survey (NHANES).

**Methods:**

We reviewed questionnaires from the 3 surveys and identified a set of comparable measures related to smoking prevalence, alcohol consumption, medical conditions, vaccination, health status, insurance coverage, cost barriers to medical care, testing for human immunodeficiency virus, and body measurements (height and weight).

We compared weighted estimates for up to 15 outcome measures, including overall measures and measures for 12 population subgroups. We produced design-appropriate point estimates and carried out statistical tests of hypotheses on the equality of such estimates. We then calculated *P* values for comparisons of NHIS and NHANES estimates with their BRFSS counterparts.

**Results:**

Although BRFSS and NHIS estimates were statistically similar for 5 of the 15 measures examined, BRFSS and NHANES estimates were statistically similar for only 1 of 6 measures. The observed differences for some of these comparisons were small, however.

**Conclusion:**

These surveys produced similar estimates for several outcome measures, although we observed significant differences as well. Many of the observed differences may have limited consequences for implementing related public health programs; other differences may require more detailed examination. In general, the range of BRFSS estimates examined here tends to parallel those from NHIS and NHANES, both of which have higher rates of participation.

## Introduction

The federal government allocates substantial resources each year for collection of state and national data to monitor trends and changes in the health of the U.S. population. Scientists, health care professionals, and policy makers use such data to understand current and emerging trends in public health, to provide a basis for the establishment and evaluation of health policies and programs, and to assess where best to apply limited public health resources. It is, therefore, imperative for health statistics and other population estimates obtained from these surveys to be of the highest possible quality.

The Behavioral Risk Factor Surveillance System (BRFSS), the world's largest ongoing random-digit–dialed (RDD) telephone survey, is conducted by the health departments in the 50 states as well as those in the District of Columbia, Puerto Rico, Guam, and the Virgin Islands, with assistance from the Centers for Disease Control and Prevention (CDC) (1). Estimates obtained from the BRFSS are based on sound methods for conducting surveys and performing statistical analyses; comparison of these estimates with those from other national surveys is important for measuring the validity and reliability of the estimates. Such comparisons are especially important because RDD surveys are facing an increasing number of newly emerging operational challenges.

The rate of response to telephone surveys has traditionally served as a proxy indicator of the survey's data quality. Link et al point out that telephone survey response rates have recently declined (2). BRFSS response rates declined between 1993 and 2006 (Figure). Several researchers have presented convincing arguments on the negative correlation between rate of nonresponse and survey data quality (3-5). RDD surveys face a unique and growing problem: an increasing number of people rely on cell phones as the only means of telephone communication. Most RDD surveys traditionally sample only from landline telephone numbers (6). Also of concern is the change in BRFSS sampling methods. As of 2004, all zero-listed telephone banks (sets of telephone numbers that include only unlisted or nonresidential numbers) were eliminated from the sampling frame to increase efficiency; this change has increased undercoverage of the population by up to 2% (7).

**Figure.**
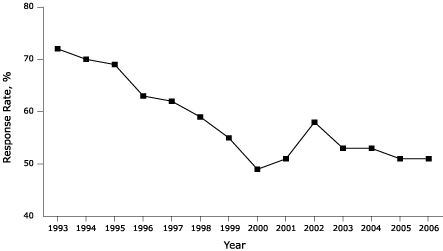
Response rates for the Behavioral Risk Factor Surveillance System, 1993–2006 (Reference 1).

**Table:** 

This study compares a number of key estimates obtained from BRFSS with related estimates from the National Health Interview Survey (NHIS) and the National Health and Nutrition Examination Survey (NHANES). These surveys have structural differences in design protocols, modes of data collection, and postsurvey procedures for data adjustment. NHIS and NHANES also have higher rates of participation than BRFSS. A comparison of the estimates from the 3 surveys can render valuable insights into the quality of the estimates. The estimates included in these comparisons are related to smoking prevalence, alcohol consumption, medical conditions, vaccination, health status, insurance coverage, cost barriers to medical care, testing for human immunodeficiency virus (HIV), and body measurements (height and weight).

Gentry and colleagues compared estimates of chronic drinking obtained from the National Institute on Alcoholism and Alcohol Abuse and estimates of current smoking obtained from NHIS with BRFSS estimates and concluded that their similarity was statistically significant (8). A more comprehensive study by Nelson et al examined the comparability of national estimates from the 1997 BRFSS and NHIS (9). Nelson et al concluded that the 2 surveys provided similar estimates for most of the overall measures examined. This research reexamines these findings after nearly a decade and makes new comparisons with estimates from NHANES and NHIS.

## Methods

### Survey designs and data

The data for this research were obtained from the 2004 public-use versions of BRFSS, NHIS, and NHANES. Although technical and methodological details of these surveys are readily available online and in print form, a brief overview of these surveys is provided here for reference purposes (10-12).

BRFSS relies on a monthly, state-based RDD sample design in which virtually all households with landline telephones have a nonzero probability of selection. States use disproportionate stratified sampling in which listed residential telephone numbers are sampled at a higher rate than unlisted residential telephone numbers. One adult aged 18 years or older is chosen at random for interview from each selected household; annual sample sizes vary from state to state. In 2004, state sample sizes ranged from 2656 in Alaska to 18,587 in Washington, with a median state-level sample size of 5903 and a weighted national response rate of 47.2%. BRFSS data from the 50 states and the District of Columbia are aggregated to provide a national sample and are weighted to reflect the sample design and to compensate for differential nonresponse and undercoverage. The resulting weights are ratio-adjusted (poststratified) within cells indexed by age and sex and, in certain states, by race/ethnicity.

NHIS is a national survey conducted annually by interviewers of the U.S. Census Bureau for the National Center for Health Statistics (NCHS) of CDC and is designed to track major trends in illness, disability, and coverage of certain health care services in the U.S. population. The annual questionnaire consists of 3 components: the family core, the sample adult core, and the sample child core. The family core collects information on all members of the household aged 17 years or older who are at home during the interview. Proxy data are collected on children and adults who are not at home. In addition, 1 sample child and 1 sample adult from each household are randomly selected, and information on each is collected with the sample child core and sample adult core questionnaires. This survey, which is conducted year-round, is based on a multistage design that starts with selection of a sample of 358 primary sampling units (PSUs) from approximately 1900 geographically designed PSUs in the country. In the second stage, all occupied houses in selected area segments in each PSU are targeted for interview. The sample for the 2004 survey consisted of 94,460 people in 36,579 households. Interviewers obtained data for the sample adult component of the questionnaire from 31,326 of the 37,388 adults eligible for the interview. The household-level response rate was 86.9%, and the conditional response rate for the sample adult component was 83.8% (calculated by dividing the number of completed sample adult interviews by the total number of eligible sample adults). Weights for NHIS data are derived from the 2000 census and adjusted by age, sex, and race/ethnicity.

NHANES, which is also conducted by NCHS, aims to assess the health and nutritional status of adults and children in the United States as a basis for setting national standards for physical measurements such as height and weight. Basic survey data are obtained in the homes of study participants; data from detailed physical examinations are obtained for a selected subset of participants at mobile examination centers. Incentives range from $20 to $100 and are given for completion of surveys and examinations and as compensation for expenses related to participating in the survey. Similar to NHIS, NHANES begins with selection of PSUs. Clusters of households are selected to be screened for specific demographic characteristics. Then a sample of eligible households is selected, and 1 or more individuals per household are interviewed. During the 2003–2004 NHANES, of the 12,761 people selected, 10,122 were interviewed, and 9643 had a physical examination. Because of the multistage design for NHANES, a complex weighting procedure is used to compensate for unequal selection probabilities and for poststratification to the Current Population Survey estimates of the U.S. population.

### Analysis

We reviewed questionnaires used for BRFSS, NHIS, and NHANES and identified a set of comparable measures related to smoking prevalence, alcohol consumption, medical conditions, vaccination, health status, insurance coverage, cost barriers to medical care, testing for HIV, and body measurements. (The exact questions used in this study are provided in the Appendix.) We identified 15 measures from NHIS (14 reported measures and 1 calculated measure, body mass index [BMI]) and 6 measures from NHANES (5 reported measures and 1 calculated measure, BMI) for comparison with their BRFSS counterparts.

We combined some response categories to create conforming scales between the 3 surveys. We also used only subsets of respondents with matching age categories for certain comparisons. For example, we grouped people with borderline diabetes with people who reported a diagnosis of diabetes. Also, we combined the response categories of fair health and poor health to create 1 category. To eliminate confounding effects attributable to different age requirements for these surveys, we excluded from analysis all respondents aged younger than 18 years. Finally, we restricted analysis of health care coverage to participants aged 18 to 64 years, and we restricted questions on influenza and pneumococcal vaccinations to participants aged 65 years or older.

A few wording differences between the 3 survey instruments could not be addressed completely by combining response categories. For instance, the 2004 BRFSS asked for the number of times during the past 30 days the person had 5 or more drinks in a single occasion, while NHIS allows a choice of unit for time (per day, week, month, or year).

To determine which estimates were statistically different, we used the SAS-callable version of SUDAAN (Research Triangle Institute, Research Triangle Park, North Carolina) to produce design-appropriate point estimates and 95% confidence intervals. We calculated corresponding *P* values for comparisons of NHIS and NHANES estimates with their BRFSS counterparts under the null hypothesis of equality of point estimates. All test statistics were produced using *z* tests for proportions for weighted data. We used a nominal α level of .05 (*P* < .05), adjusted for multiple comparisons, to determine significant differences between 2 corresponding surveys.

In addition to comparing overall estimates, we compared the following 12 demographic categories:

- Age: 18 to 34, 35 to 54, and 55 years or older.
- Education: less than a high school diploma, high school diploma or some college, and college diploma or more.
- Sex: male or female.
- Race/ethnicity: Hispanic, non-Hispanic white, non-Hispanic black, and non-Hispanic other.

## Results

The data show varying levels of agreement between the 3 surveys (Tables 1–4). BRFSS and NHIS overall estimates were statistically similar for 5 of the 15 items examined: current smoker, diabetes, BMI, average number of alcoholic drinks per occasion, and no health insurance. BRFSS and NHANES estimates were statistically similar for 1 of the 6 items analyzed: self-reported height. BRFSS and NHIS were significantly different for 10 items; for 9 of these, BRFSS estimates were significantly higher than NHIS estimates. Only 1 BRFSS estimate (for binge drinking) was significantly lower than the NHIS estimate. In contrast, BRFSS estimates were lower than NHANES estimates for 4 of the 5 items for which differences were found; diabetes was the sole exception.

The magnitude of differences varied widely. For example, the relative difference between BRFSS and NHIS estimates of height is less than 1% (derived by dividing the difference [0.14 inches] between the BRFSS and NHIS overall point estimates by the NHIS overall point estimate [66.96 inches]). For weight, the relative difference is 1.2% (2.13%/170.90%). The relative difference in overall estimates of ever having smoked a cigarette is 4.0% (1.68%/42.35%) and of having received an influenza vaccination in the past 12 months, 4.6% (2.95%/64.63%). At the other end of the spectrum, however, the relative differences in BRFSS and NHIS estimates are 94.4% (7.35%/7.79%) for experiencing cost barriers to medical care, 35.1% (3.48%/9.91%) for having asthma, 33.9% (4.16%/12.28%) for reporting poor or fair health, 26.4% (9.15%/34.61%) for having ever had an HIV test, and 11.5% (6.54%/56.84%) for having ever had a pneumonia vaccination. In contrast, the estimate for binge drinking is 8.3% (0.39 drinks/4.70 drinks) lower for BRFSS than for NHIS.

As a relative percentage of the NHANES estimates, BRFSS estimates show less variation. For diabetes, BRFSS estimates are 31.3% (1.92%/6.14%) higher, but they are 12.2% (2.89%/23.60%) lower for current smoking, 8.3% (3.96%/47.99%) lower for ever having smoked a cigarette, 2.1% lower for BMI (0.58/27.55), and 2.0% (3.55 lb/176.58 lb) lower for self-reported weight.

BRFSS and NHIS estimates for diabetes prevalence were similar for all 12 population subgroups. The following tabulation shows the number of subgroups for which BRFSS and NHIS estimates were statistically similar:

**Table:** 

**Survey Question**	**No. of Subgroups Similar/No. Subgroups Surveyed **
No health insurance	10/12
Binge drinking	8/12
BMI	7/12
Influenza vaccination	6/9
Number of alcohol drinks	5/12
Current smoker	5/12
Self-reported height	3/12
Pneumonia vaccination	2/9
Self-reported weight	2/12
Ever smoked	2/12

The estimates for fair or poor health status, asthma, having been tested for HIV, and not receiving medical care because of cost were significantly different between all subgroups examined.

Similarity between BRFSS and NHANES estimates among the 12 subgroups was greatest for self-reported height (9 subgroups), followed by diabetes (8 subgroups), current smoker (6 subgroups), self-reported weight (5 subgroups), BMI (4 subgroups), and ever having smoked a cigarette (2 subgroups).

The estimates provided by the 3 surveys for the 4 demographic characteristics (age, sex, race/ethnicity, and education) are not entirely consistent despite being indexed to population totals; estimates for educational attainment differ most notably.

## Discussion

Some of the estimates from the 2004 BRFSS, NHIS, and the 2003–2004 NHANES vary overall and within certain subgroups. While a number of these differences are small and may not warrant more examinations, other differences — especially those that reflect a relative difference of more than 20% — may require further investigations. The BRFSS estimates tended to be higher than NHIS estimates but lower than NHANES estimates. For the 6 items examined in all 3 surveys, 1 of the BRFSS estimates (self-reported height) was identical to NHANES, 2 fell between those of NHIS and NHANES (ever smoke cigarettes and self-reported weight), and 3 were statistically identical to NHIS (current smoking status, diabetes, and BMI).

The extent of similarity between the 2004 BRFSS and NHIS estimates was less than what was reported by Nelson and colleagues (9) for the 1997 comparisons. They found statistically significant differences between overall estimates for 6 of 14 items examined; BRFSS estimates were lower for current smoking, height, and BMI and higher for pneumococcal vaccination, cost as a barrier to medical care, and reporting fair or poor health.

Several explanations may account for the inconsistencies

 BRFSS estimates and estimates from the other 2 surveys, including 1) differences in questionnaire wording, 2) mode of survey administration, 3) sampling design differences and related postsurvey adjustments such as weighting procedures, and 4) use of incentives or proxy data.

Differences in questionnaire wording represent some of the greatest challenges in comparing survey results. The wording of the BRFSS and NHIS questionnaires was identical for only 4 of the items examined (ever smoked cigarettes, ever had a pneumonia vaccination, health status, and asthma) and for 1 of the NHANES items (ever smoked cigarettes). Some questions that seemed to be measuring the same concept differed in ways that may have led to differences in their final estimates. For example, the questions used to assess binge drinking are dissimilar in wording, time frame, and response categories. BRFSS asks, "Considering all types of alcoholic beverages, how many times during the past 30 days did you have 5 or more drinks on an occasion?"; NHIS asks, "In the past year, on how many days did you have 5 or more drinks of any alcoholic beverages?" Although both surveys attempt to measure the same concept, differences in wording and time frame could explain the small but statistically significant differences in estimates that we observed.

Even with identical wording, however, different modes of survey administration can produce varying results and magnify differences in measurement (13,14). BRFSS is a telephone survey, whereas NHIS and NHANES are conducted face-to-face. For BRFSS the interaction is aural and limited to voice communication, whereas NHIS and NHANES rely on both aural and visual cues. Such differences in the patterns of interviewer–respondent communication and situational contexts may explain some of the variation in survey estimates.

Differences in weighting adjustment to compensate for design-imposed differential selection probabilities may also explain some of the observed differences in survey estimates. The comparison of weighted estimates for basic demographic characteristics, such as race/ethnicity and education, shows that even when weights are applied, differences remain between survey respondents. These differences may lead, in part, to some of the inconsistencies in health and risk estimates between the 3 surveys. In addition, the 3 surveys use different sources to obtain population control totals for weight calibrations. BRFSS uses population estimates from Claritas (Claritas Inc, San Diego, California), a private data vendor that uses census projections as part of its process for developing yearly population estimates, whereas NHIS uses census projections and NHANES relies on the Current Population Survey estimates. These sources for population control totals vary in their analysis of the U.S. population by sex, age, and race/ethnicity. In addition, the weighting procedures for both NHIS and NHANES account for differences in the race/ethnicity distribution in each state, whereas BRFSS includes this type of adjustment in selected states only. Education was the socioeconomic indicator that differed most markedly between the 3 surveys. BRFSS is revising its weighting methodology to account for educational differences; the new weight will be applied to public release data sets in 2010 (15).

Other factors, perhaps minor, may also contribute to differences in survey estimates. For instance, BRFSS and NHIS do not offer any incentives for survey participation, but NHANES relies on incentives to secure higher rates of response. The use of incentives may alter a participant's survey response or attract a different mix of participants (16). Also, differences between BRFSS and NHIS estimates may be affected by the use of proxy data in NHIS. Although BRFSS does not allow proxy respondents, some NHIS estimates (e.g., cost as a barrier to medical care, health status) can be based on proxy data. BRFSS and NHIS provide significantly different estimates for both of these measures. Hays et al report that agreement between self and proxy responses are often good for measures of function that are directly observable but poor for subjective measures (17). Moreover, Nelson and colleagues suggest that proxy-derived data are reliable for demographic and body measures but are less reliable for questions on medications and alcohol consumption (18).

Although the many differences in design, procedures, and context of these surveys may confound our ability to single out the proximal causes of differences in survey estimates noted in this research, the comparison of estimates for similar concepts is important. These periodic comparisons are particularly important because BRFSS, NHIS, and NHANES are critical components of the U.S. public health system, with each providing essential data for policy makers, researchers, and the public alike. The periodic examination of when and why measurements of similar concepts vary is equally important.

These research findings also need to be interpreted in light of tangible implications. Although many of the estimates examined here differed statistically, their programmatic implications and impact on actionable policy are subject to debate. What constitutes a "significant" difference is ultimately up to the people who use the data. Perhaps the good news for BRFSS is that despite declines in survey participation rates during the past decade, BRFSS estimates do not appear to be radically different from similar estimates produced by NHIS and NHANES. In fact, for the 6 common items in these surveys, BRFSS estimates were either similar to NHIS or NHANES estimates or were found between the estimates of these 2 surveys.

## Figures and Tables

**Table 1:** Summary Results From 6 Questions Common to BRFSS, NHIS, and NHANES Among Respondents Aged 18 or Older

Question/Characteristic	BRFSS	NHIS		NHANES	
**Ever smoked cigarettes**	**% (95% CI)**	**% (95% CI)**	***z* Test (*P*)^a^**	**% (95% CI)**	***z* Test (*P*)^a^**
Overall	44.0 (43.7-44.4)	42.4 (41.6-43.1)	4.08 (<.001)	48.0 (45.8-50.2)	−3.79 (<.001)
Aged 18-34 y	36.8 (36.1-37.5)	33.2 (31.9-34.4)	4.88 (<.001)	42.1 (37.3-47.1)	−2.30 (.02)
Aged 35-54 y	44.8 (44.2-45.3)	43.5 (42.3-44.6)	2.05 (.04)	49.2 (46.5-51.9)	−3.39 (.001)
Aged ≥55 y	50.9 (50.4-51.5)	50.6 (49.4-51.8)	0.50 (.62)	55.6 (51.9-59.1)	−2.70 (.007)
Male	49.9 (49.4-50.5)	48.3 (47.3-49.4)	2.66 (.008)	53.4 (50.0-56.8)	−2.14 (.03)
Female	38.5 (38.1-38.9)	36.8 (35.9-37.8)	3.21 (.001)	42.4 (39.7-45.2)	−3.00 (.003)
White non-Hispanic	48.0 (47.6-48.3)	47.0 (46.1-47.8)	2.05 (.04)	50.6 (48.9-52.2)	−3.18 (.001)
Black non-Hispanic	36.7 (35.6-37.8)	33.3 (31.6-35.1)	3.26 (.001)	34.6 (27.7-42.2)	0.61 (.54)
Hispanic	32.5 (31.2-33.8)	28.2 (26.6-29.9)	4.01 (<.001)	42.8 (36.9-48.9)	−3.55 (<.001)
Other non-Hispanic	36.9 (34.9-38.9)	30.2 (27.3-33.3)	3.61 (<.001)	48.2 (35.7-60.9)	−1.84 (.06)
<High school diploma	49.8 (48.6-51.0)	46.6 (45.0-48.3)	3.07 (.002)	58.8 (51.0-66.1)	−2.48 (.01)
High school diploma or some college	48.0 (47.5-48.5)	45.7 (44.7-46.8)	3.95 (<.001)	55.2 (50.1-60.3)	−3.01 (.003)
≥College diploma	34.8 (34.2-35.4)	35.7 (34.6-36.8)	−1.41 (.16)	43.0 (40.2-45.8)	−6.01 (<.001)
**Currently smoke cigarettes**	**% (95% CI)**	**% (95% CI)**	***z* Test (*P*)^a^**	**% (95% CI)**	***z* Test (*P*)^a^**
Overall	20.7 (20.4-21.0)	20.9 (20.3-21.6)	−0.64 (.52)	23.6 (21.4-25.9)	−2.70 (.007)
Aged 18-34 y	25.2 (24.5-25.8)	23.6 (22.5-24.8)	2.35 (.02)	30.3 (26.8-34.1)	−2.90 (.004)
Aged 35-54 y	23.3 (22.8-23.7)	23.8 (22.9-24.8)	−1.07 (.28)	24.5 (20.4-29.1)	−0.59 (.55)
Aged ≥55 y	12.7 (12.4-13.1)	14.2 (13.4-15.0)	−3.41 (.001)	10.8 (9.3-12.4)	2.68 (.007)
Male	23.0 (22.6-23.5)	23.5 (22.7-24.4)	−0.93 (.35)	25.8 (22.8-29.1)	−1.85 (.06)
Female	18.5 (18.2-18.8)	18.6 (17.8-19.3)	−0.10 (.92)	21.3 (18.5-24.4)	−2.01 (.04)
White non-Hispanic	21.4 (21.0-21.7)	22.3 (21.6-23.1)	−2.23 (.03)	23.6 (21.1-26.3)	−1.77 (.07)
Black non-Hispanic	21.9 (21.0-22.9)	20.4 (18.8-22.1)	1.55 (.12)	21.0 (16.1-26.9)	0.36 (.72)
Hispanic	16.8 (15.8-17.8)	15.0 (13.8-16.3)	2.19 (.03)	27.3 (22.3-32.9)	−4.12 (<.001)
Other non-Hispanic	19.8 (18.3-21.4)	16.5 (14.0-19.5)	2.00 (.04)	21.7 (15.0-30.2)	−0.52 (.60)
<High school diploma	29.1 (28.1-30.2)	26.8 (25.3-28.4)	2.39 (.02)	33.3 (29.0-38.0)	−1.93 (.05)
High school diploma or some college	24.4 (24.0-24.8)	24.1 (23.3-25.0)	0.67 (.50)	30.6 (26.1-35.6)	−2.79 (.005)
≥College diploma	10.8 (10.5-11.2)	13.6 (12.7-14.4)	−5.90 (<.001)	18.9 (16.9-21.1)	−7.99 (<.001)
**Ever told have diabetes**	**% (95% CI)**	**% (95% CI)**	***z* Test (*P*)^a^**	**% (95% CI)**	***z* Test (*P*)^a^**
Overall	8.1 (7.9-8.2)	8.1 (7.8-8.5)	−0.20 (.84)	6.1 (5.1-7.4)	3.45 (.001)
Aged 18-34 y	1.4 (1.3-1.6)	1.5 (1.3-1.8)	−0.62 (.54)	1.1 (0.6-2.1)	0.97 (.33)
Aged 35-54 y	6.4 (6.2-6.7)	6.4 (5.9-7.0)	0.07 (.95)	5.9 (4.6-7.6)	0.76 (.45)
Aged ≥55 y	17.2 (16.8-17.7)	17.3 (16.4-18.2)	−0.10 (.92)	16.6 (13.5-20.2)	0.39 (.70)
Male	8.3 (8.0-8.6)	8.3 (7.8-8.8)	0.17 (.87)	5.2 (4.2-6.4)	5.62 (<.001)
Female	7.8 (7.6-8.0)	7.9 (7.5-8.4)	−0.51 (.61)	7.1 (5.9-8.6)	1.03 (.30)
White non-Hispanic	7.6 (7.5-7.8)	7.7 (7.3-8.1)	−0.09 (.93)	5.8 (4.5-7.5)	2.57 (.01)
Black non-Hispanic	11.4 (10.7-12.2)	10.7 (9.8-11.8)	1.11 (.27)	8.4 (6.8-10.3)	3.42 (.001)
Hispanic	7.6 (7.0-8.4)	8.2 (7.3-9.1)	−0.91 (.36)	6.1 (3.7-9.8)	1.08 (.28)
Other non-Hispanic	8.5 (7.4-9.8)	8.5 (6.7-10.8)	0.01 (.99)	5.8 (2.6-12.1)	1.29 (.20)
<High school diploma	13.0 (12.2-13.8)	12.4 (11.5-13.4)	0.94 (.35)	12.2 (9.5-15.7)	0.50 (.62)
High school diploma or some college	8.3 (8.0-8.5)	8.0 (7.6-8.5)	0.83 (.41)	6.3 (5.0-7.8)	3.12 (.002)
≥College diploma	5.7 (5.4-6.0)	6.1 (5.6-6.7)	−1.30 (.20)	4.7 (3.6-6.2)	1.66 (.10)
**Self-reported height**	**Inches (95% CI)**	**Inches (95% CI)**	***z* Test (*P*)^a^**	**Inches (95% CI)**	***z* Test (*P*)^a^**
Overall	67.1 (67.1-67.1)	67.0 (66.9-67.0)	3.88 (<.001)	67.1 (66.8-67.4)	0 (>.99)
Aged 18-34 y	67.5 (67.4-67.5)	67.3 (67.2-67.4)	2.34 (.02)	67.4 (67.2-67.7)	0.22 (.82)
Aged 35-54 y	67.3 (67.3-67.4)	67.1 (67.0-67.2)	4.25 (<.001)	67.4 (67.0-67.7)	−0.23 (.82)
Aged ≥55 y	66.4 (66.4-66.5)	66.4 (66.3-66.4)	1.30 (.19)	65.9 (65.6-66.2)	3.61 (<.001)
Male	70.0 (70.0-70.1)	69.9 (69.9-70.0)	3.33 (.001)	69.8 (69.5-70.0)	2.20 (.03)
Female	64.3 (64.3-64.3)	64.2 (64.2-64.3)	3.13 (.002)	64.4 (64.1-64.6)	−0.50 (.62)
White non-Hispanic	67.5 (67.4-67.5)	67.2 (67.2-67.3)	6.10 (<.001)	67.4 (67.2-67.7)	0.23 (.82)
Black non-Hispanic	67.1 (67.0-67.2)	67.0 (66.9-67.2)	0.74 (.46)	67.1 (66.9-67.3)	0.18 (.86)
Hispanic	65.5 (65.3-65.6)	65.7 (65.6-65.9)	−2.93 (.003)	65.1 (64.6 -65.5)	1.81 (.07)
Other non-Hispanic	66.4 (66.2-66.6)	65.4 (65.1-65.6)	5.75 (<.001)	66.1 (65.6-66.6)	1.14 (.26)
<High school diploma	65.8 (65.7-66.0)	66.1 (66.0-66.2)	−2.93 (.003)	66.0 (65.6-66.4)	−0.79 (.43)
High school diploma or some college	67.0 (67.0-67.0)	67.0 (66.9-67.0)	1.34 (0.18)	67.1 (66.8-67.4)	−0.66 (.51)
≥College diploma	67.7 (67.7-67.8)	67.3 (67.3-67.4)	7.83 (<.001)	67.4 (67.0-67.7)	2.16 (.03)
**Self-reported weight**	**Pounds (95% CI)**	**Pounds (95% CI)**	***z* Test (*P*)^a^**	**Pounds (95% CI)**	***z *Test (*P*)^a^**
Overall	173.0 (172.7-173.3)	170.9 (170.4-171.5)	6.71 (<.001)	176.6 (174.2-178.9)	−3.20 (.001)
Aged 18-34 y	168.5 (167.9-169.1)	166.3 (165.3-167.4)	3.60 (<.001)	171.5 (167.4-175.5)	−1.55 (.12)
Aged 35-54 y	178.0 (177.5-178.4)	174.6 (173.7-175.5)	6.59 (<.001)	183.2 (180.6-185.9)	−4.16 (<.001)
Aged ≥55 y	171.4 (171.0-171.9)	170.8 (169.9-171.7)	1.17 (.24)	174.7 (171.7-177.6)	−2.31 (.02)
Male	191.0 (190.6-191.5)	189.2 (188.5-189.9)	4.36 (<.001)	192.4 (190.2-194.6)	−1.27 (.20)
Female	155.3 (155.0-155.6)	153.4 (152.8-154.1)	4.96 (<.001)	160.3 (156.6-164.0)	−2.85 (.004)
White non-Hispanic	173.7 (173.4-174.0)	171.3 (170.6-171.9)	6.60 (<.001)	176.7 (174.0-179.3)	−2.39 (.02)
Black non-Hispanic	183.0 (182.0-184.0)	178.9 (177.4-180.4)	4.41 (<.001)	189.9 (183.8-196.0)	−2.40 (.02)
Hispanic	166.5 (165.5-167.6)	168.0 (166.8-169.3)	−1.80 (.07)	168.9 (164.9-172.9)	−1.22 (.22)
Other non-Hispanic	161.3 (159.8-162.9)	152.6 (150.3-154.9)	6.16 (<.001)	158.7 (148.4-169.0)	0.53 (.60)
<High school diploma	170.6 (169.6-171.5)	168.9 (167.7-170.1)	2.05 (.04)	174.2 (170.0-178.4)	−1.80 (.07)
High school diploma or some college	174.4 (174.0-174.8)	172.2 (171.4-173.0)	4.66 (<.001)	181.0 (178.1-183.9)	−4.78 (<.001)
≥College diploma	171.6 (171.1-172.1)	170.0 (169.2-170.9)	3.08 (.002)	175.3 (172.0-178.6)	−2.35 (.02)
**BMI (calculated from height and weight)**	**BMI (95% CI)**	**BMI (95% CI)**	***z *Test (*P*)^a^**	**BMI (95% CI)**	***z* Test (*P*)^a^**
Overall	27.0 (26.9-27.0)	27.0 (26.9-27.1)	−1.12 (.26)	27.6 (27.2-27.9)	−3.20 (.001)
Aged 18-34 y	26.0 (25.9-26.1)	26.0 (25.8-26.2)	0.32 (.75)	26.4 (25.9 -27.0)	−1.69 (.09)
Aged 35-54 y	27.5 (27.5-27.6)	27.6 (27.4-27.7)	−0.39 (.69)	28.4 (28.0 -28.8)	−4.01 (<.001)
Aged ≥55 y	27.2 (27.2-27.3)	27.4 (27.3-27.5)	−2.39 (.02)	28.2 (27.7-28.7)	−4.31 (<.001)
Male	27.4 (27.4-27.5)	27.4 (27.4-27.6)	−0.34 (.73)	27.8 (27.5-28.1)	−2.70 (.007)
Female	26.5 (26.4-26.6)	26.6 (26.5-26.7)	−1.31 (.19)	27.3 (26.6-28.0)	−2.43 (.015)
White non-Hispanic	26.7 (26.7-26.7)	26.8 (26.7-26.9)	−2.04 (.04)	27.3 (26.8-27.8)	−2.58 (.01)
Black non-Hispanic	28.6 (28.4-28.7)	28.7 (28.4-28.9)	−0.54 (.59)	29.8 (29.0-30.6)	−3.19 (.001)
Hispanic	27.6 (27.4-27.8)	27.6 (27.4-27.8)	−0.30 (.77)	28.1 (27.4-28.8)	−1.43 (.15)
Other non-Hispanic	25.7 (25.4-26.0)	24.7 (24.4-25.0)	4.47 (<.001)	25.4 (24.1-26.7)	0.41 (.68)
<High school diploma	27.9 (27.8-28.1)	27.5 (27.3-27.7)	3.27 (.001)	28.2 (27.5-28.8)	−0.83 (.41)
High school diploma or some college	27.2 (27.2-27.3)	27.3 (27.2-27.4)	−1.19 (.23)	28.2 (27.9-28.5)	−6.77 (<.001)
≥College diploma	26.2 (26.1-26.2)	26.4 (26.3-26.5)	−3.88 (<.001)	27.1 (26.6-27.7)	−3.74 (<.001)

BRFSS indicates Behavioral Risk Factor Surveillance System; NHIS, National Health Interview Survey; NHANES, National Health and Nutrition Examination Survey; CI, confidence interval; BMI, body mass index.

a *P* values represent comparison with BRFSS.

**Table 2:** Summary Results From 5 Questions Common to BRFSS and NHIS Among Respondents Aged 18 or Older

**Question/Characteristic**	BRFSS	NHIS	Statistic
**Fair or poor health status**	**% (95% CI)**	**% (95% CI)**	***z *Test (*P*)**
Overall	16.4 (16.2-16.7)	12.3 (11.8-12.7)	15.45 (<.001)
Aged 18-34 y	9.1 (8.6-9.6)	4.5 (4.0-5.0)	13.37 (<.001)
Aged 35-54 y	14.7 (14.2-15.1)	10.5 (9.9-11.1)	10.92 (<.001)
Aged ≥55 y	26.7 (26.2-27.2)	22.9 (22.0-23.9)	6.97 (<.001)
Male	15.2 (14.7-15.6)	11.4 (10.8-12.0)	10.19 (<.001)
Female	17.6 (17.3-18.0)	13.1 (12.5-13.7)	12.64 (<.001)
White non-Hispanic	13.7 (13.5-14.0)	11.4 (10.9-12.0)	7.78 (<.001)
Black non-Hispanic	19.9 (19.0-20.8)	18.0 (16.6-19.5)	2.15 (.03)
Hispanic	27.9 (26.7-29.2)	13.0 (11.9-14.1)	17.57 (<.001)
Other non-Hispanic	14.1 (12.8-15.4)	9.3 (7.4-11.8)	3.64 (<.001)
<High school diploma	39.0 (37.8-40.2)	25.9 (24.5-27.4)	13.86 (<.001)
High school diploma or some college	16.5 (16.2-16.9)	11.9 (11.3-12.5)	13.29 (<.001)
≥College diploma	7.3 (7.0-7.6)	6.1 (5.6-6.7)	3.73 (<.001)
**Ever told have asthma**	**% (95% CI)**	**% (95% CI)**	***z *Test (*P*)**
Overall	13.4 (13.1-13.6)	9.9 (9.5-10.3)	14.09 (<.001)
Aged 18-34 y	15.3 (14.8-15.9)	10.3 (9.6-11.1)	10.41 (<.001)
Aged 35-54 y	12.8 (12.4-13.2)	9.6 (9.0-10.2)	9.07 (<.001)
Aged ≥55 y	12.2 (11.8-12.5)	9.9 (9.3-10.6)	5.69 (<.001)
Male	11.6 (11.3-12.0)	8.5 (7.9-9.1)	8.64 (<.001)
Female	15.0 (14.7-15.4)	11.2 (10.7-11.8)	11.53 (<.001)
White non-Hispanic	13.5 (13.2-13.8)	10.2 (9.7-10.8)	11.18 (<.001)
Black non-Hispanic	14.8 (14.0-15.6)	11.3 (10.2-12.6)	4.63 (<.001)
Hispanic	11.8 (11.0 -12.7)	7.5 (6.6-8.5)	6.71 (<.001)
Other non-Hispanic	14.0 (12.7-15.5)	7.7 (6.1-9.7)	5.42 (<.001)
<High school diploma	14.3 (13.5-15.1)	10.7 (9.8-11.8)	5.39 (<.001)
High school diploma or some college	13.6 (13.2-13.9)	10.0 (9.4-10.7)	9.96 (<.001)
≥College diploma	12.8 (12.4-13.2)	9.5 (8.9-10.1)	8.55 (<.001)
**Ever had HIV test**	**% (95% CI)**	**% (95% CI)**	***z *Test (*P*)**
Overall	43.8 (43.4-44.2)	34.6 (33.9-35.3)	21.51 (<.001)
Aged 18-34 y	51.6 (50.8-52.4)	43.9 (42.5-45.3)	9.56 (<.001)
Aged 35-54 y	44.1 (43.5-44.7)	40.6 (39.4-41.8)	5.24 (<.001)
Aged ≥55 y	23.6 (22.9-24.5)	16.5 (15.6-17.4)	11.73 (<.001)
Male	41.2 (40.6-41.9)	31.7 (30.7-32.7)	15.96 (<.001)
Female	46.2 (45.7-46.8)	37.3 (36.4-38.3)	16.06 (<.001)
White non-Hispanic	40.3 (39.9-40.7)	31.5 (30.6-32.4)	17.95 (<.001)
Black non-Hispanic	62.6 (61.4-63.9)	51.4 (49.5-53.2)	9.87 (<.001)
Hispanic	45.9 (44.4-47.30	38.2 (36.5-39.9)	6.80 (<.001)
Other non-Hispanic	43.3 (41.1-45.6)	32.7 (29.5-36.1)	5.21 (<.001)
<High school diploma	41.8 (40.3-43.3)	30.7 (29.1-32.4)	9.84 (<.001)
High school diploma or some college	43.1 (42.6-43.7)	32.8 (31.8-33.7)	18.32 (<.001)
≥College diploma	45.6 (44.9-46.3)	39.2 (38.0-40.3)	9.72 (<.001)
**Binge drinking past 30 days**	**% (95% CI)**	**% (95% CI)**	***z *Test (*P*)**
Overall	4.3 (4.2-4.4)	4.7 (4.5-4.9)	−2.91 (.004)
Aged 18-34 y	4.2 (4.1-4.4)	4.2 (3.9-4.5)	0.51 (.61)
Aged 35-54 y	4.1 (4.0-4.3)	5.0 (4.6-5.4)	−3.97 (<.001)
Aged ≥55 y	5.3 (4.9-5.8)	6.2 (5.3-7.1)	−1.80 (.07)
Male	4.7 (4.6-4.9)	5.0 (4.8-5.3)	−1.86 (.06)
Female	3.0 (3.0-3.2)	3.6 (3.2-3.9)	−2.42 (.02)
White non-Hispanic	4.3 (4.2-4.4)	4.7 (4.5-5.0)	−2.75 (.006)
Black non-Hispanic	4.8 (4.2-5.3)	5.7 (4.8-6.6)	−1.73 (.08)
Hispanic	3.8 (3.5-4.1)	4.1 (3.7-4.6)	−1.07 (.28)
Other non-Hispanic	5.3 (4.3-6.4)	4.2 (2.6-5.8)	1.19 (.23)
<High school diploma	5.1 (4.6-5.6)	5.8 (5.1-6.5)	−1.55 (.12)
High school diploma or some college	4.6 (4.4-4.8)	4.9 (4.6-5.3)	−1.73 (.08)
≥College diploma	3.4 (3.3-3.6)	3.8 (3.4-4.1)	−1.97 (.05)
**Average number of alcoholic drinks per occasion**	**No. (95% CI)**	**No. (95% CI)**	***z *Test (*P*)**
Overall	2.4 (2.4-2.5)	2.5 (2.4-2.5)	−1.06 (.29)
Aged 18-34 y	3.2 (3.1-3.3)	3.2 (3.1-3.3)	0 (>.99)
Aged 35-54 y	2.3 (2.2-2.3)	2.3 (2.2-2.3)	−0.71 (.48)
Aged ≥55 y	1.7 (1.6-1.7)	1.8 (1.7-1.8)	−2.85 (.004)
Male	2.9 (2.8-2.9)	2.9 (2.8-3.0)	−0.80 (.42)
Female	1.9 (1.9-1.9)	1.9 (1.9-2.0)	−2.68 (.007)
White non-Hispanic	2.3 (2.3-2.3)	2.4 (2.3-2.4)	−3.79 (<.001)
Black non-Hispanic	2.3 (2.2-2.4)	2.4 (2.2-2.5)	−0.74 (.46)
Hispanic	3.4 (3.2-3.6)	3.0 (2.9-3.2)	3.33 (.001)
Other non-Hispanic	2.6 (2.4-2.8)	2.2 (2.1-2.4)	2.39 (.02)
<High school diploma	3.6 (3.4-3.8)	3.1 (3.0-3.3)	3.52 (<.001)
High school diploma or some college	2.6 (2.6-2.6)	2.7 (2.6-2.8)	−1.57 (.12)
≥College diploma	2.0 (1.9-2.0)	2.0 (2.0-2.1)	−2.12 (.03)

BRFSS indicates Behavioral Risk Factor Surveillance System; NHIS, National Health Interview Survey; CI, confidence interval; HIV, human immunodeficiency virus.

**Table 3:** Summary Results From 2 Questions Common to BRFSS and NHIS Among Respondents Aged 18 to 64 years

**Question/Characteristic**	BRFSS% (95% CI)	NHIS% (95% CI)	*z *Test (*P*)
**No medical care due to cost**			
Overall	15.1 (14.8-15.4)	7.8 (7.4-8.2)	30.36 (<.001)
Aged 18-34 y	17.6 (17.0-18.2)	7.6 (7.0-8.4)	21.52 (<.001)
Aged 35-54 y	14.6 (14.2-15.0)	8.0 (7.5-8.6)	19.52 (<.001)
Aged 55-64 y	10.9 (10.4-11.5)	7.4 (6.6-8.3)	6.78 (<.001)
Male	13.0 (12.5-13.4)	6.8 (6.3-7.4)	17.34 (<.001)
Female	17.3 (16.9-17.7)	8.7 (8.2-9.3)	25.51 (<.001)
White non-Hispanic	12.7 (12.4-13.0)	7.6 (7.1-8.0)	18.98 (<.001)
Black non-Hispanic	20.3 (19.4-21.3)	9.7 (8.8-10.8)	15.11 (<.001)
Hispanic	21.7 (20.6-22.9)	8.3 (7.5-9.3)	17.84 (<.001)
Other non-Hispanic	16.2 (14.8-17.8)	4.8 (3.6-6.3)	11.07 (<.001)
<High school diploma	27.6 (26.4-28.9)	11.4 (10.4-12.6)	18.75 (<.001)
High school diploma or some college	16.5 (16.1-16.9)	8.8 (8.3-9.3)	22.09 (<.001)
≥College diploma	8.4 (8.0-8.8)	5.0 (4.6-5.5)	10.85 (<.001)
**No health insurance coverage**			
Overall	18.7 (18.4-19.1)	19.3 (18.7-20.0)	−1.67 (.09)
Aged 18-34 y	25.9 (25.2-26.6)	27.0 (25.9-28.2)	−1.67 (.10)
Aged 35-54 y	15.4 (15.0-15.9)	15.8 (15.1-16.6)	−0.93 (.35)
Aged 55-64 y	11.3 (10.8-11.9)	11.7 (10.6-12.9)	−0.65 (.52)
Male	20.3 (19.7-20.8)	21.3 (20.4-22.2)	−1.77 (.08)
Female	17.2 (16.8-17.6)	17.5 (16.8-18.2)	−0.64 (.52)
White non-Hispanic	13.6 (13.3-13.9)	14.1 (13.4-14.7)	−1.29 (.20)
Black non-Hispanic	23.2 (22.2-24.3)	22.9 (21.2-24.7)	0.34 (.73)
Hispanic	37.2 (35.8-38.6)	42.8 (41.0-44.5)	−4.87 (<.001)
Other non-Hispanic	19.2 (17.6-21.0)	20.5 (17.5-23.9)	−0.68 (.50)
<High school diploma	43.6 (42.2-45.2)	41.1 (39.2-43.0)	2.10 (.04)
High school diploma or some college	19.8 (19.4-20.3)	20.4 (19.6-21.2)	−1.10 (.27)
≥College diploma	8.1 (7.7-8.5)	8.8 (8.0-9.6)	−1.51 (.13)

BRFSS indicates Behavioral Risk Factor Surveillance System; NHIS, National Health Interview Survey; CI, confidence interval.

**Table 4:** Summary Results From 2 Questions Common to BRFSS and NHIS Among Respondents Aged 65 years or Older

**Question/**Characteristic	BRFSS% (95% CI)	NHIS% (95% CI)	*z *Test(*P*)
**Had an influenza vaccination in the past 12 months**			
Overall	67.6 (66.9-68.2)	64.6 (63.2-66.0)	3.64 (<.001)
Male	68.9 (67.7-70.0)	64.1 (61.9-66.2)	3.81 (<.001)
Female	66.6 (65.8-67.5)	65.0 (63.3-66.7)	1.68 (.09)
White non-Hispanic	70.2 (69.6-70.9)	67.3 (65.8-68.9)	3.36 (.001)
Black non-Hispanic	49.6 (46.5-52.7)	45.4 (41.0-49.8)	1.54 (.12)
Hispanic	57.9 (53.6-62.0)	54.6 (49.3-59.7)	0.97 (.33)
Other non-Hispanic	65.0 (58.4-71.0)	62.2 (50.0-72.9)	0.42 (.68)
<High school diploma	61.5 (59.7-63.2)	58.7 (56.0-61.4)	1.69 (.09)
High school diploma or some college	68.0 (67.2-68.9)	65.0 (62.8-67.1)	2.61 (.009)
≥College diploma	71.0 (69.6-72.4)	71.1 (68.4-73.7)	−0.08 (.94)
**Ever had a pneumonia vaccination**			
Overall	63.4 (62.7-64.1)	56.8 (55.3-58.3)	7.66 (<.001)
Male	61.8 (60.6-63.0)	54.3 (51.7-56.8)	5.30 (<.001)
Female	64.5 (63.6-65.4)	58.7 (56.9-60.6)	5.46 (<.001)
White non-Hispanic	66.9 (66.3-67.6)	60.9 (59.3-62.5)	6.67 (<.001)
Black non-Hispanic	43.0 (40.0-46.1)	38.5 (34.0-43.2)	1.61 (.11)
Hispanic	46.9 (42.4-51.5)	33.7 (29.0-38.9)	3.85 (<.001)
Other non-Hispanic	53.7 (46.5-60.8)	37.4 (27.9-48.0)	2.58 (.01)
<High school diploma	55.2 (53.3-57.0)	49.3 (46.5-52.0)	3.46 (.001)
High school diploma or some college	65.1 (64.2-66.0)	58.5 (56.2-60.8)	5.23 (<.001)
≥College diploma	65.5 (64.0-66.9)	62.9 (59.9-65.8)	1.54 (.12)

BRFSS indicates Behavioral Risk Factor Surveillance System; NHIS, National Health Interview Survey; CI, confidence interval.

## Appendix

**Table:** Comparison of Questions Used in Study of Population Estimates Generated by 3 National Health Surveys: BRFSS, NHIS, and NHANES

Domain	BRFSS	Comparison Question^a^
Ever smoked	Have you smoked at least 100 cigarettes in your entire life?	—
Current smoker	—	NHIS: Do you now smoke cigarettes every day, some days, or not at all? NHANES: Do you now smoke cigarettes every day or some days?
Diabetes	Have you ever been told by a doctor that you have diabetes?	NHIS: Other than during pregnancy, have you EVER been told by a doctor or health care professional that you have diabetes or sugar diabetes? NHANES: Other than during pregnancy, have you ever been told by a doctor or health care professional that you have diabetes or sugar diabetes?
Influenza vaccination	During the past 12 months, have you had a flu shot?	NHIS: During the past 12 months, have you had a flu shot? A flu shot is usually given in the fall and protects against influenza for the flu season.
Pneumonia vaccination	Have you ever had a pneumonia shot? This shot is usually given only once or twice in a person's lifetime and is different from the flu shot. It is also called the pneumococcal vaccine.	—
No health insurance coverage	Do you have any kind of health care coverage, including health insurance, prepaid plans such as HMOs, or government plans such as Medicare?	NHIS: I have recorded you are (covered by/not covered by) health insurance. Is this correct?
Do not receive medical care due to cost	Was there a time in the past 12 months when you needed to see a doctor but could not because of cost?	NHIS: During the past 12 months, was there any time when [you/someone in the family] needed medical care, but did not get it because [you/the family] couldn't afford it?
Health status	Would you say that in general your health is excellent, very good, good, fair, or poor?	—
Asthma	Have you ever been told by a doctor, nurse, or other health professional that you had asthma?	—
Testing for human immunodeficiency virus	Have you ever been tested for HIV? Do not count tests you may have had as part of a blood donation.	NHIS: The next questions are about the test for HIV (the virus that causes AIDS). Except for tests you may have had as part of blood donations, have you ever been tested for HIV?
Height	About how tall are you without shoes?	NHIS: Total height in inches. NHANES: Standing height (cm).
Weight	About how much do you weigh without shoes?	NHIS: How much do you weigh without shoes? NHANES: Weight (kg).
Average number of alcoholic drinks per occasion	On the days when you drank, about how many drinks did you drink on the average? (response range: 1-76)	NHIS: In the past year, on those days that you drank alcoholic beverages, on the average, how many drinks did you have?
Binge drinking	Considering all types of alcoholic beverages, how many times during the past 30 days did you have 5 or more drinks on an occasion? (response range: 1-76)	NHIS**:** In the past year, on how many days did you have 5 or more drinks of any alcoholic beverages? NHANES: How many days per week, per month, or per year did you have 5 or more drinks on a single day?

BRFSS indicates Behavioral Risk Factor Surveillance System; NHIS, National Health Interview Survey; NHANES, National Health and Nutrition Examination Survey.

a Text of questions for NHIS is shown only when different from BRFSS.
